# Florzolotau (18F) retention is linked to neuropsychological performance in tauopathy

**DOI:** 10.1002/alz.71319

**Published:** 2026-04-01

**Authors:** Atsushi Shimizu, Yu Iwabuchi, Shogyoku Bun, Memi Watanabe, Masahito Kubota, Sho Shimohama, Toshiki Tezuka, Keisuke Takahata, Hajime Tabuchi, Morinobu Seki, Yuki Momota, Yasuharu Yamamoto, Ryo Shikimoto, Yu Mimura, Shin Kurose, Ryosuke Sakurai, Toshiki Takayama, Yuka Hoshino, Takayuki Hoshino, Natsumi Suzuki, Ayaka Morimoto, Azusa Osumi, Masaru Mimura, Masahiro Jinzaki, Daisuke Ito

**Affiliations:** ^1^ Department of Radiology Keio University School of Medicine Tokyo Japan; ^2^ Department of Neuropsychiatry Keio University School of Medicine Tokyo Japan; ^3^ Department of Neurology Keio University School of Medicine Tokyo Japan; ^4^ Advanced Neuroimaging Center, Institute for Quantum Medical Science, National Institutes for Quantum Science and Technology Chiba Japan; ^5^ Department of Psychiatry and Behavioral Science, Graduate School of Medicine Juntendo University Tokyo Japan; ^6^ Memory Center Keio University School of Medicine Tokyo Japan

**Keywords:** frontotemporal lobar degeneration, neuropsychological test, Statistical Parametric Mapping, tau positron emission tomography

## Abstract

**INTRODUCTION:**

Florzolotau (18F) positron emission tomography visualizes three‐ and four‐repeat tau isoforms. We aimed to evaluate correlation of tau tracer retention with neuropsychological performance across the Alzheimer's disease (AD) continuum and non‐AD tauopathies.

**METHODS:**

This study enrolled 178 patients with cognitive impairment and 60 volunteers. Participants were divided into AD continuum (*n* = 120) and non‐AD tauopathy (*n* = 98) groups and assessed using the Mini‐Mental State Examination (MMSE), Clinical Dementia Rating, Functional Activity Questionnaire, Wechsler Memory Scale‐Revised Logical Memory, Alzheimer's Disease Assessment Scale Cognitive subscale, Trail Making Test (TMT), word fluency, and the Japanese Adult Reading Test. Voxel‐based analysis was performed.

**RESULTS:**

In the AD continuum group, all cognitive test performances significantly correlated with tracer uptake, particularly in the left cortical areas. In the non‐AD tauopathy group, strong correlations were observed with MMSE and TMT Part B performance.

**DISCUSSION:**

Neuropsychological performance and regional tau pathology distribution are correlated in patients with tauopathy, with differences between AD continuum and non‐AD tauopathy.

**CLINICAL TRIAL REGISTRATION:**

This trial is registered with UMIN Clinical Trials (UMIN‐CTR number: 000032027); submitted for registration on March 30, 2018; first patient enrolment was on July 3, 2018.

## Highlights

- Florzolotau (18‐F) retention is linked to neuropsychological deficits in tauopathies.
- In the Alzheimer's disease (AD) continuum, posterior cortical tau retention is linked to cognitive decline.
- In non‐AD tauopathies, frontoparietal tau retention is linked to specific cognitive skills.

## 1 BACKGROUND

Tau, a microtubule‐associated protein, plays a crucial role in microtubule assembly and stabilization.^1^ Pathological accumulation of misfolded tau is common across neurodegenerative disorders known as tauopathies, including Alzheimer's disease (AD) and various forms of frontotemporal lobar degeneration (FTLD).^2^ AD, the most prevalent tauopathy, accounts for 60% to 80% of dementia cases globally.^3^ Clinically, AD is characterized by gradual cognitive decline, with memory impairment, language difficulties, and executive dysfunction as hallmark symptoms.^4^ Pathologically, it is marked by senile plaques and neurofibrillary tangles (NFTs), resulting from amyloid beta (Aβ) and tau pathology, respectively.^5^ FTLD encompasses a range of heterogeneous pathologies, characterized by progressive atrophy of the frontal and/or temporal lobes. Its major subdivisions are defined by the type of accumulated proteins. Approximately half of FTLD cases are tauopathies, including FTLD with Pick bodies, frontotemporal dementia, and Parkinsonism linked to chromosome 17, corticobasal degeneration, and progressive supranuclear palsy (PSP).^6^

Braak staging describes the spread of NFTs in AD, starting with binding in the medial temporal lobe and spreading to neocortical regions.^7^, ^8^ In a tau positron emission tomography (PET) study, Brown et al. investigated tau magnitude and extent (Tau‐MaX), which resembled Braak stages and served as a marker of AD severity.^9^ The intensity and topography of baseline tau PET images were found to correlate with future atrophy using voxel‐based analysis, which also accounts for the magnitude and extent of tau burden.^10^ Although there has been interest in exploring regional tau burden, few studies have examined both the spatial spread and intensity of tau.

In AD, the pathological condition is triggered by the cascade of amyloid pathology; however, histopathological studies indicate that the extent of this deposition is not a reliable predictor of dementia severity.^11^ In contrast, cognitive impairment severity correlates more closely with the burden of neocortical NFTs, which consist of hyperphosphorylated tau proteins. *Post mortem* studies have demonstrated the presence of NFTs in the medial temporal lobe in cognitively normal older adults and those with cognitive impairments, a condition termed primary age‐related tauopathy (PART).^12^, ^13^ Moreover, PET studies have shown that flortaucipir (18F) tau uptake is more strongly correlated with neuropsychological test scores, compared to amyloid PET uptake, in the AD spectrum.^14^

Early tau PET tracers had limitations, such as off‐target binding and low signal‐to‐noise ratios, restricting their use primarily to AD. However, second‐generation tracers, such as florzolotau (18F), have overcome many of these issues. Furthermore, florzolotau (18F) enables the detection of various tau conformers, including three‐repeat and/or four‐repeat tau, which are present in primary tauopathies, thus facilitating the assessment of AD and non‐AD tauopathies.^15^

We aimed to investigate the florzolotau (18F) retention patterns and their correlation with neuropsychological test scores in patients with the AD continuum and non‐AD tauopathies, as well as to examine the differences between these patient populations.

## 2 METHODS

### 2.1 Participants and clinical measurements

This study enrolled 178 patients with cognitive impairment and 60 healthy volunteers who visited the memory clinic at Keio University Hospital between July 3, 2018 and November 30, 2023. Patients with dementia were clinically diagnosed with AD; FTLD subtypes, including PSP, corticobasal syndrome (CBS), behavioral‐variant frontotemporal dementia, and progressive non‐fluent aphasia; dementia with Lewy bodies/Parkinson's disease (DLB/PD); mild cognitive impairment (MCI); traumatic brain injury (TBI); and mental disorders. All participants underwent structural magnetic resonance imaging (MRI), amyloid PET with 18F‐florbetaben (FBB), and tau PET with 18F‐PI‐2620 or florzolotau (18F) within 6 months. The inclusion criteria and the exclusion and discontinuance criteria are described in Tables S1 and S2 in supporting information, respectively. AD was defined based on a positive amyloid PET scan, with healthy controls being amyloid PET negative. Cognitively normal volunteers who were positive for amyloid PET were classified as having preclinical AD. Non‐AD tauopathy was defined as dementia with negative amyloid PET scan but positive tau PET scan.

**RESEARCH IN CONTEXT**

1. **Systematic review**: The authors reviewed literature regarding tau positron emission tomography and cognition. Tau pathology correlates with cognitive decline in Alzheimer's disease (AD), and florzolotau (18F) is vital for visualizing both three‐ and four‐repeat tau isoforms in non‐AD tauopathies.
2. **Interpretation**: In this study, we investigated the correlation between florzolotau (18F) retention and detailed neuropsychological assessments in 178 patients with cognitive impairment, classified into AD continuum and non‐AD tauopathy groups. In the AD continuum group, tracer retention in posterior cortical areas strongly correlated with performance across cognitive domains. Conversely, the non‐AD tauopathy group exhibited distinct correlation patterns, particularly in the frontal and parietal lobes. These findings validate florzolotau (18F) as a sensitive biomarker for tracking domain‐specific cognitive impairment across the tauopathy spectrum.
3. **Future directions**: Future research should focus on longitudinal studies to assess the predictive value of florzolotau (18F) retention. Additionally, neuropathological confirmation is necessary to validate the binding specificity for heterogeneous non‐AD tauopathies.

### 2.2 Neuropsychological tests

Neuropsychological tests were conducted to assess cognitive function and psychiatric symptoms, including the Geriatric Depression Scale (GDS), Mini‐Mental State Examination (MMSE), Clinical Dementia Rating (CDR), Functional Activity Questionnaire (FAQ), Wechsler Memory Scale‐Revised Logical Memory (LM) for immediate and delayed recall, Alzheimer's Disease Assessment Scale Cognitive subscale (ADAS‐Cog), Trail Making Test (TMT) Parts A (TMT‐A) and B (TMT‐B), Word Fluency (WF‐C: category and ‐I: initial), and Japanese Adult Reading Test (JART). These assessments were performed following standard administration procedures, as outlined in a previous study.^16^ Additionally, an item‐by‐item analysis of the MMSE and ADAS‐Cog was conducted to evaluate specific cognitive domains.

### 2.3 Structural MRI

Three‐dimensional T1‐weighted imaging (3D BRAVO, repetition time = 6.8 ms, echo time = 3.0 ms, field of view = 23.0 mm, voxel size = 0.9 × 0.9 × 1.0 mm, and flip angle = 8°) was performed using a Discovery MR750 3.0‐T scanner (GE Healthcare).

### 2.4 Amyloid PET

All participants underwent 18F‐FBB PET using 18F‐FBB, which was manufactured at our hospital following good manufacturing practice, using an automated synthesizer (Synthera V2; IBA). PET imaging was performed as previously described.^17^ After image acquisition and reconstruction, a nuclear medicine expert assessed the image quality according to the Neuraceq guidelines.^18^

### 2.5 Tau PET

The PET scan protocol using florzolotau (18F) has been previously described.^15^ PET data were reconstructed using the PMOD Neuro tool (PMOD Technologies) for visual tau PET imaging, which was then fitted to the structural MRI anatomically. The standardized uptake value ratio (SUVR) was calculated using the whole cerebellum as the reference and the SUVR images were fused with the MRI images. Tau tracer retention was assessed visually through consensus among neurologists, radiologists, and psychiatrists specializing in dementia and neuroimaging. The results were initially categorized as positive or negative. In positive patients, the images were interpreted based on regional tracer uptake patterns that were either consistent with a negative AD tau pattern or AD tau pattern. An AD tau pattern consisted of increased neocortical activity in the posterolateral temporal or occipital regions. In patients with negative AD tau pattern, tau deposition patterns were evaluated to identify disease‐specific patterns, as outlined in the previous report.^15^

### 2.6 Statistical analysis

The demographic characteristics of patients were compared between the AD continuum and non‐AD tauopathy groups using a chi‐squared test for categorical data and a two‐tailed unpaired *t* test for numeric data. *P* values < 0.05 were considered statistically significant.

Tau PET imaging data were pre‐processed and analyzed using statistical parametric mapping software (https://www.fil.ion.ucl.ac.uk/spm/software/spm12/) running within the Matlab R2023a software environment (MathWorks). First, the individual PET and MRI images were converted to Neuroimaging Informatics Technology Initiative format. The PET images were then re‐oriented to place the origin at the anterior commissure and coregistered to the MRI images. These coregistered PET images were warped into the Montreal Neurological Institute space and converted to SUVR images, using the whole cerebellum as the reference. The normalized SUVR image was smoothed with an 8 × 8 × 8 mm Gaussian filter. Linear regression analyses, adjusted for age and sex, were performed to assess the correlations between tau tracer uptake and neuropsychological test scores. The initial voxel threshold was set to 0.001, uncorrected for multiple comparisons, and a corrected *P* value for the cluster size of *P* < 0.05 was obtained using the family‐wise error method. A cluster size exceeding the expected threshold of voxels per cluster was considered significant.

## 3 RESULTS

### 3.1 Participant characteristics

**FIGURE 1:**
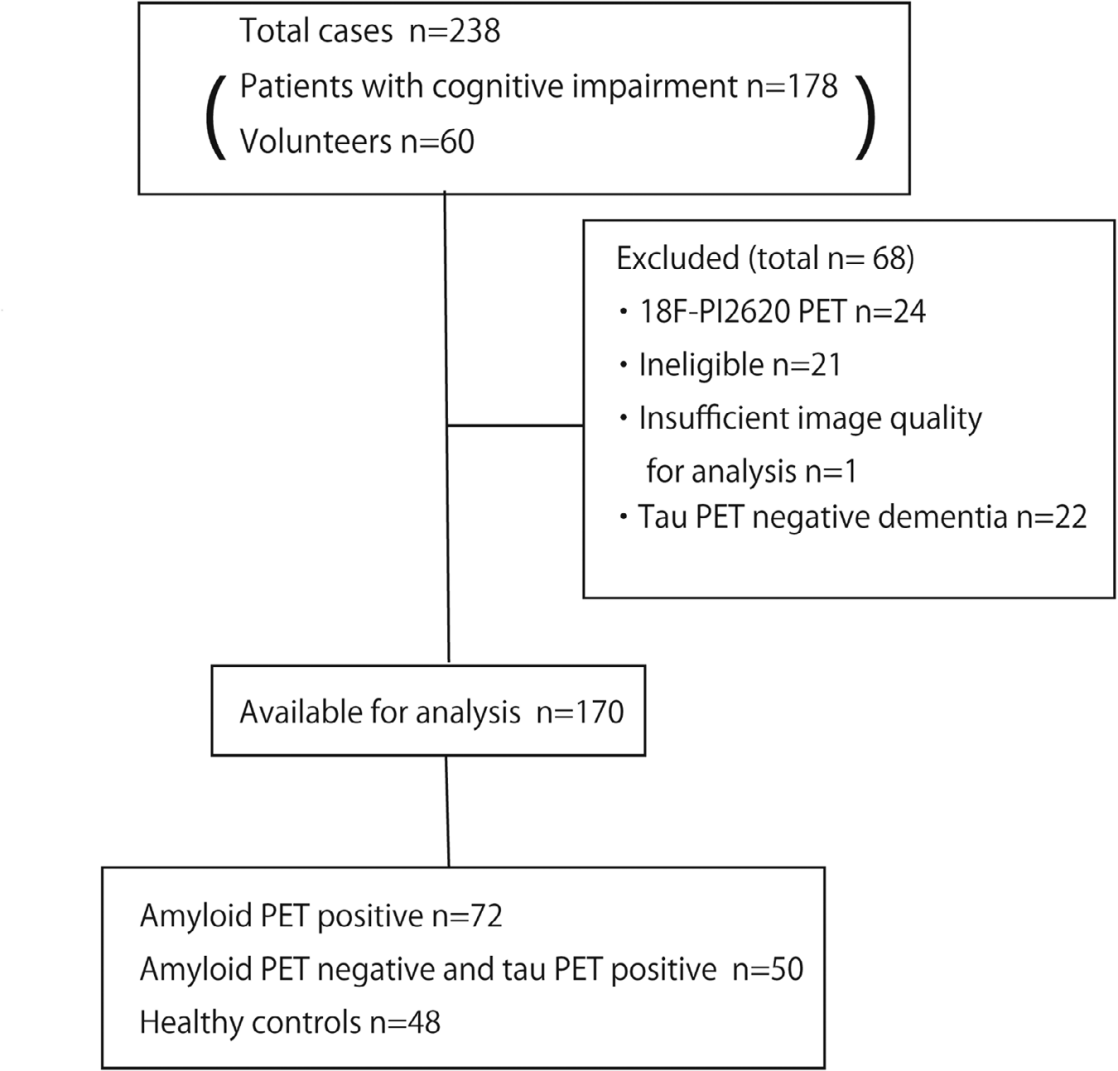
Flow diagram of participant inclusion. PET, positron emission tomography.

**FIGURE 2:**
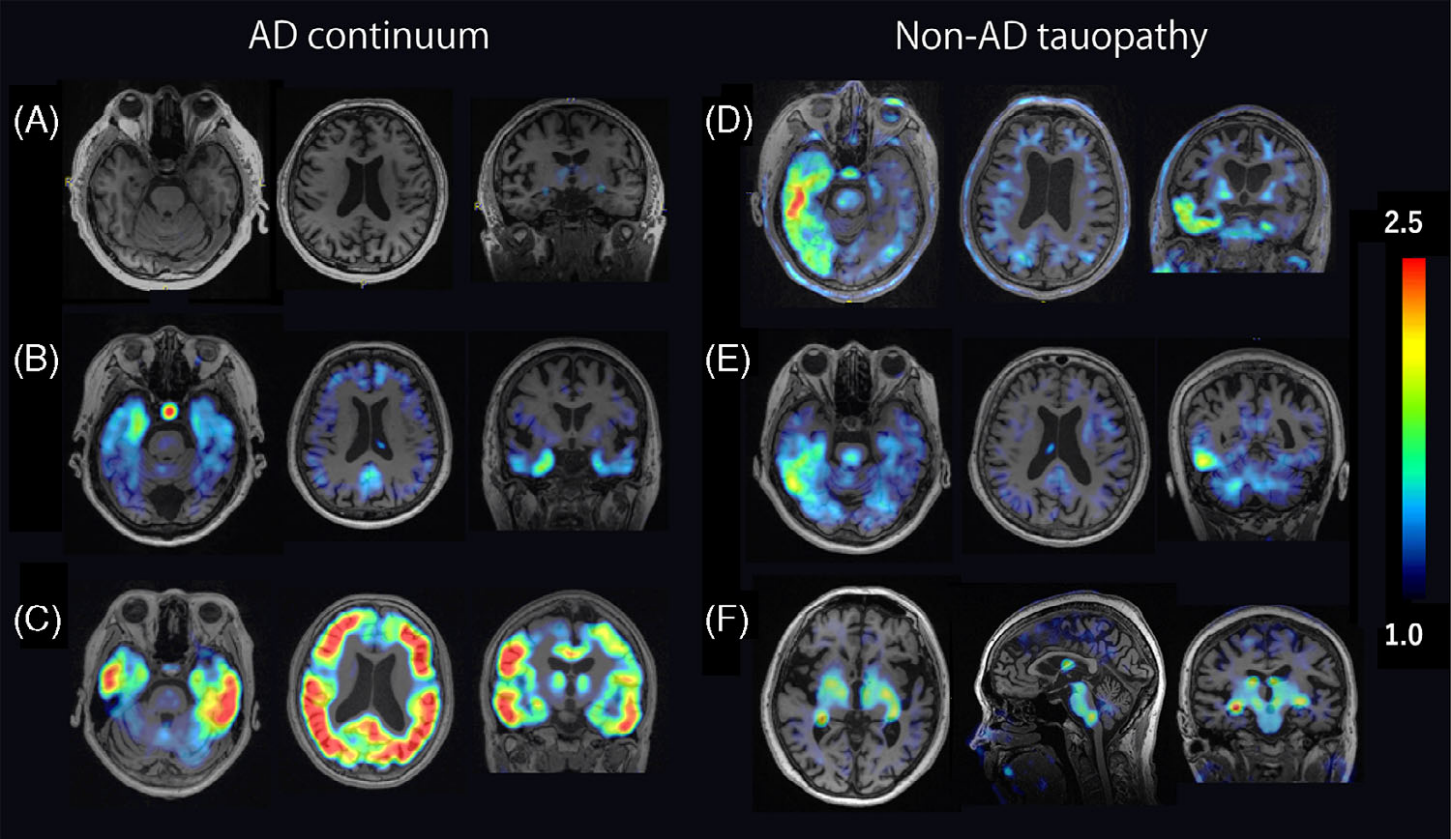
Tau PET images of representative patients. Tau PET images of: (A) a 60‐year‐old male HC participant, showing no intense tau tracer uptake; (B) a 70‐year‐old female patient with MCI due to AD, showing mild tau tracer uptake in the bilateral temporal lobes; (C) a 50‐year‐old female patient with AD, showing intense tau tracer uptake in the neocortex; (D) a 70‐year‐old male patient with bvFTD, showing increased tau tracer uptake in the right temporal lobe; (E) a 60‐year‐old female patient with SD, showing increased tau tracer uptake in the right temporal lobe; (F) a 60‐year‐old female patient with PSP, showing increased tau tracer uptake in the midbrain and subthalamic nucleus. AD, Alzheimer's disease; bvFTD, behavioral variant frontotemporal dementia; HC, healthy control; MCI, mild cognitive impairment; PET, positron emission tomography; PSP, progressive supranuclear palsy; SD, semantic dementia.

Figure 1 illustrates the study flow diagram. A total of 68 participants were excluded: 24 owing to receipt of 18F‐PI‐2620, 21 based on various criteria (including 3 with a gap of more than 6 months between MRI and PET imaging, 1 with loss of communication, 3 with a GDS score > 6, 1 with < 12 years of education, 4 who withdrew consent, 4 with clinic visit issues, 1 with a history of hematopoietic cell transplantation, 1 with a brain tumor, 1 with subdural hematoma, 1 with bipolar disorder, and 1 who was unable to undergo psychological tests), 22 with tau PET‐negative dementia (12 with MCI due to non‐tauopathy, 5 with FTLD due to non‐tauopathy, 3 with depression, 2 with unknown dementia), and 1 owing to data failure. Finally, 170 participants were enrolled in the study. The AD continuum group (*n* = 120) comprised 72 amyloid PET‐positive participants (including 10 with preclinical AD) and 48 healthy controls. The non‐AD tauopathy group (*n* = 98) comprised 17 participants with MCI of unknown etiology (including 2 volunteers), 14 with FTLD, 6 with CBS, 4 with mental disorders (including 2 each with paranoid disorder and depression), 5 with TBI, 3 with PSP, 1 with DLB/PD, and 48 healthy controls. The demographic characteristics of the participants are summarized in Table 1. Significant differences between the groups were observed in age, and the GDS, LM‐immediate, and LM‐delayed scores. Figure 2 shows the tau PET images of representative patients from the AD continuum and non‐AD tauopathy groups.

**TABLE 1:** Participant characteristics and neuropsychological test performance.

	Patients with AD (*n* = 72)	Patients with non‐AD tauopathy (*n* = 50)	*P* value	Healthy controls (*n* = 48)	Patients with non‐tauopathy dementias (*n* = 22)
Age (years)	72.2 ± 9.1	68.5 ± 11.4	0.021	68.5 ± 8.0	67.0 ± 11
Sex (male)	35/72	28/49	0.356	25/48	12/22
Education (years)	14.5 ± 1.9	14.8 ± 1.8	0.319	15.1 ± 2.4	14.3 ± 2.1
GDS score	2.72 ± 2.6	5.36 ± 3.90	<0.001	1.96 ± 1.8	3.44 ± 3.6
MMSE score	23.6 ± 5.2	25.5 ± 5.5	0.064	29.1 ± 1.0	25.9 ± 4.5
CDR‐G score	0.49 ± 0.29	0.42 ± 0.33	0.319	0 ± 0	0.39 ± 0.30
CDR‐Ss score	1.82 ± 1.9	1.56 ± 2.2	0.490	0.020 ± 0.01	1.34 ± 1.8
FAQ score	3.22 ± 3.6	2.73 ± 3.9	0.485	0.33 ± 0.90	2.59 ± 3.8
LM‐immediate score	5.10 ± 3.7	7.65 ± 4.7	0.001	13.3 ± 2.7	7.68 ± 5.1
LM‐delay score	2.82 ± 3.7	5.50 ± 4.7	<0.001	12.6 ± 3.0	6.32 ± 5.0
ADAS‐Cog score	12.3 ± 7.5	10.1 ± 11.0	0.201	3.83 ± 1.8	8.97 ± 8.8
WF‐C score	28.8 ± 11	29.0 ± 11.6	0.940	42.6 ± 7.3	32.3 ± 13
WF‐I score	22.4 ± 9.1	19.1 ± 8.19	0.047	27.5 ± 8.7	22.1 ± 12
TMT‐A score	86.1 ± 67	66.9 ± 38.4	0.077	49.5 ± 17	76.2 ± 52.5
TMT‐B score	162.4 ± 119	141 ± 102	0.348	82.7 ± 32	138 ± 80
JART score	30.0 ± 10.0	28.9 ± 12.2	0.855	36.7 ± 9.3	32.5 ± 1.5

*Note*: Data are presented as means ± standard deviations or numbers of participants.

Abbreviations: AD, Alzheimer's disease; ADAS‐Cog, Alzheimer's Disease Assessment Scale Cognitive subscale; CDR‐G, Clinical Dementia Rating Global score; CDR‐S, Clinical Dementia Rating Sum of Boxes; FAQ, Functional Activity Questionnaire; GDS, Geriatric Depression Scale; JART, Japanese Adult Reading Test; LM, Wechsler Memory Scale‐Revised Logical Memory; MMSE, Mini‐Mental State Examination; TMT‐A, Trail Making Test Part A; TMT‐B, Trail Making Test Part B; WF, Word Fluency (C: category, I: initial).

### 3.2 Correlation between neuropsychological scores and tau PET uptake in the AD continuum group

**FIGURE 3:**
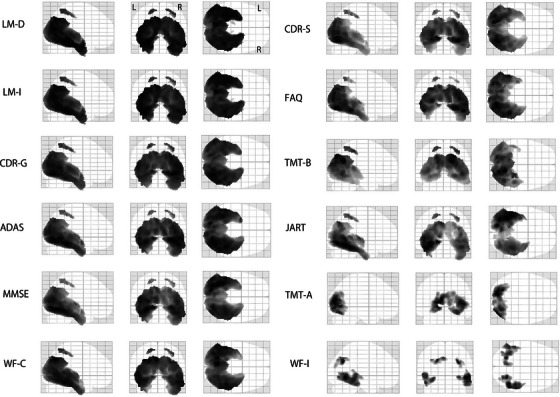
Voxel‐wise correlation between tau tracer retention and neuropsychological performance in the AD continuum group. Voxel‐wise regression analyses show a significant correlation of tau tracer retention with poorer performance on all neuropsychological tests. Regions in black represent voxel clusters with significant correlations. AD, Alzheimer's disease; ADAS, Alzheimer's Disease Assessment Scale Cognitive subscale; CDR‐G, Clinical Dementia Rating Global score; CDR‐S, Clinical Dementia Rating Sum of Boxes; FAQ, Functional Activity Questionnaire; JART, Japanese Adult Reading Test; LM, Wechsler Memory Scale‐Revised Logical Memory; MMSE, Mini‐Mental State Examination; TMT‐A, Trail Making Test Part A; TMT‐B, Trail Making Test Part B; WF, Word Fluency (C: category, I: initial).

Significant correlations between florzolotau (18F) uptake and cognitive test performance were observed in the AD continuum group (Table 2). Figure 3 presents the results of voxel‐based analysis, highlighting regions in black that represent voxel clusters which exhibited significant correlations. Increased florzolotau (18F) uptake correlated with poorer performance on multiple neuropsychological tests, including the ADAS‐Cog, CDR, FAQ, JART, LM‐immediate, LM‐delayed, MMSE, TMT‐A, TMT‐B, WF‐C, and WF‐I. Tau tracer retention was primarily noted in posterior cortical areas, specifically the temporal, occipital, and parietal lobes. LM performance showed the strongest correlation with the largest cluster (28,425 voxels). Additionally, tau retention in the right temporal lobe correlated with ADAS‐Cog performance, that in the left temporal lobe with JART performance, and that in the right parietal lobe with TMT‐B performance. Tau retention in the precuneus was significantly correlated with performance on the ADAS‐Cog, CDR, FAQ, JART, LM‐immediate, LM‐delayed, MMSE, TMT‐B, and WF‐C.

**TABLE 2:** Sizes of the clusters in the brain demonstrating the highest correlation with neuropsychological test performance.

Neuropsychological test	AD continuum group	Non‐AD tauopathy group
LM‐delay	28,425	–
LM‐immediate	28,210	130
CDR‐G	27,875	–
ADAS‐Cog	27,130	–
MMSE	26,782	1857
WF‐C	25,888	121
CDR‐S	24,683	–
FAQ	23,332	–
TMT‐B	21,149	7988
JART	19,691	–
TMT‐A	5662	–
WF‐I	1689	–

*Note*: The number of the most highly correlated clusters are presented. A higher value indicates a larger area.

Abbreviations: AD, Alzheimer's disease; ADAS‐Cog, Alzheimer's Disease Assessment Scale Cognitive subscale; CDR‐G, Clinical Dementia Rating Global score; CDR‐S, Clinical Dementia Rating Sum of Boxes; FAQ, Functional Activity Questionnaire; GDS, Geriatric Depression Scale; JART, Japanese Adult Reading Test; LM, Wechsler Memory Scale‐Revised Logical Memory; MMSE, Mini‐Mental State Examination; TMT‐A, Trail Making Test Part A; TMT‐B, Trail Making Test Part B; WF, Word Fluency (C: category, I: initial).

Tables 3 and 4 and Figures S1 and S2 in supporting information present a detailed breakdown of the correlations of individual items of the ADAS‐Cog and MMSE. Performance in the word recall, follow commands, and word recognition tasks of the ADAS‐Cog, as well as those in the calculation and follow commands tasks of the MMSE, demonstrated strong correlations with tracer retention in the left temporal lobes within the AD continuum group. Performance in the orientation task of the ADAS‐Cog and registration and delayed recall tasks of the MMSE correlated with tracer retention in the bilateral temporal lobes. Performance in the repeat sentence task of the MMSE was associated with tracer retention in the left parietal and temporal lobes. Performance in the constructional praxis task of the ADAS‐Cog correlated with tracer retention in the left occipital and parietal lobes, whereas that in the ideational praxis task of the ADAS‐Cog was linked to tracer retention in the right occipital and parietal lobes.

**TABLE 3:** Alzheimer's Disease Assessment Scale Cognitive subscale items and the most highly correlated region of the cerebrum.

	AD continuum group	Non‐AD tauopathy group
Word recall	Left temporal lobe Ke = 27,269	–
Naming objects and fingers	–	Left frontal and temporal lobes Ke = 4227
Language	–	–
Constructional praxis	Left occipital and parietal lobes Ke = 3610	–
Comprehension of spoken language	–	Left frontal lobe Ke = 33
Ideational praxis	Right occipital and parietal lobes Ke = 5802	–
Word finding difficulty	Right occipital lobe Ke = 482	–
Orientation	Bilateral temporal lobes Ke = 25793	–
Follow commands	Left temporal lobe Ke = 2362	Left frontal lobe Ke = 49
Word recognition	Left temporal lobe Ke = 18174	
Remembering test instructions	–	Left frontal lobe Ke = 43

*Note*: Ke is the number of the most highly correlated clusters. A higher Ke value indicates a larger area.

Abbreviation: AD, Alzheimer's disease.

**TABLE 4:** Mini‐Mental State Examination items and the most highly correlated region of the cerebrum.

	AD continuum group	Non‐AD tauopathy group
Orientation time	Left temporal lobe Ke = 23,921	–
Orientation place	Left temporal lobe Ke = 21,607	Left frontal and right temporal lobes Ke = 396
Immediate recall	–	–
Calculation	Left temporal lobe Ke = 8110	–
Follow commands	Left temporal lobe Ke = 5971	Right occipital lobe Ke = 521
Reading	–	Left frontal lobe Ke = 392
Writing	Left occipital lobe Ke = 170	–
Copy design	Left occipital lobe Ke = 657	Right temporal lobe Ke = 1469
Registration	Bilateral temporal lobes Ke = 26263	Left frontal and temporal lobes Ke = 448
Delayed recall	Bilateral temporal lobes Ke = 25512	Left frontal lobe Ke = 86
Naming	Left temporal lobe Ke = 116	–
Sentence repetition	Left parietal and temporal lobes Ke = 4850	Bilateral temporal lobes Ke = 8058

*Note*: Ke is the number of the most highly correlated clusters. A higher Ke value indicates a larger area.

Abbreviation: AD, Alzheimer's disease.

### 3.3 Correlation between neuropsychological scores and tau PET retention in the non‐AD tauopathy group

**FIGURE 4:**
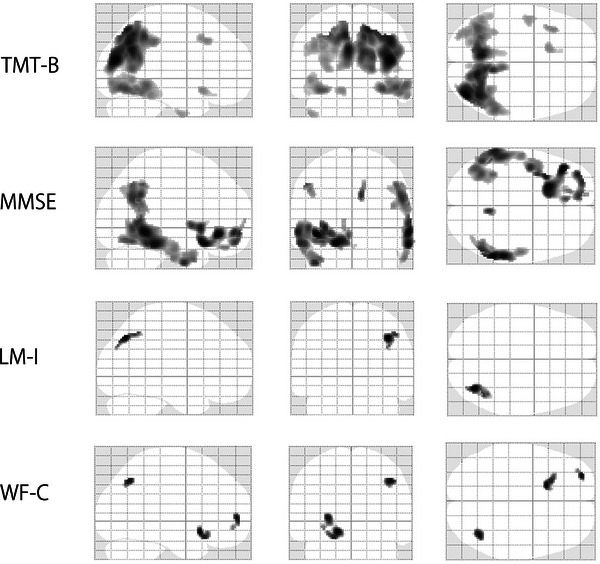
Voxel‐wise correlation between tau tracer retention and neuropsychological performance in the non‐AD tauopathy group. Voxel‐wise regression analyses show a significant correlation of tau tracer retention with poorer performance on LM‐I, MMSE, TMT‐B, and WF‐C. Regions in black represent voxel clusters with significant correlations. LM‐I, Wechsler Memory Scale‐Revised Logical Memory: immediate; MMSE, Mini‐Mental State Examination; TMT‐B, Trail Making Test Part B; WF‐C, Word Fluency: category.

Figure 4 presents the voxel‐based analysis results for the non‐AD tauopathy group.

WF‐C performance was significantly correlated with tau tracer retention in the right parietal lobe and left frontal lobe. TMT‐B performance showed a correlation with tracer retention in the bilateral parietal lobes, whereas LM‐immediate performance was associated with tracer retention in the right parietal lobe. Tau pathology in the left frontal and bilateral temporal lobes correlated with the MMSE scores.

As shown in Tables 3 and 4 and Figures S1 and S2, the performance on the task of naming objects and fingers of the ADAS‐Cog showed strong correlations with tau tracer retention in the left frontal and temporal lobes. The MMSE copy design task performance correlated with tracer retention in the right temporal lobe, whereas the MMSE repeat sentence task performance correlated with tracer retention in the bilateral temporal lobes.

## 4 DISCUSSION

Our findings indicated that increased tau tracer retention is correlated with impaired cognitive function in patients with tau‐induced dementia. Neuropsychological tests revealed distinct spatial tau distribution patterns and differences between the AD continuum and non‐AD tauopathy groups. Tau deposition is known to disrupt synaptic function and initiate cognitive decline. Previous autopsy studies demonstrated that dementia severity is positively related to the number of NFTs formed by tau protein in the neocortex.^19^ Tau PET studies have similarly shown strong relationships between 18F‐AV‐1451 uptake and cognitive impairment in patients with AD.^20^, ^21^, ^22^ In the present study, significant correlations were observed in the posterior cortical areas, including the temporal, parietal, and occipital lobes, within the AD continuum group. A previous tau PET study also reported a similar retention pattern in patients with MCI or early AD.^23^ This tau distribution pattern aligns with the pathological model of tau spreading beyond the medial temporal lobe into the neocortex, as described in Braak staging for AD.^24^ When the neocortex is involved, patients typically experience declines in cognitive and social function. Our findings corroborate the findings of previous tau PET imaging studies, reinforcing this characteristic pathological pattern. This study contributes to understanding the tau pathology underlying domain‐specific cognitive performance and supports the potential of tau PET as a biomarker for tracking tau‐induced dementia.

Several neuropsychological tests showed strong correlations with tau tracer retention. Among them, LM, widely used to assess memory function,^25^ showed the strongest correlation with tau retention in the AD continuum group. Our findings revealed a predominant distribution in the temporal lobe, including the entorhinal cortex and hippocampus, regions critical to memory.^26^ No significant difference was observed between the LM‐immediate and LM‐delayed scores. The TMT is commonly used in clinical neuropsychological assessment of executive function.^27^ TMT‐A is useful for assessing visual perception, processing speed, and spatial attention, whereas TMT‐B assesses working memory, set‐shifting ability, and cognitive flexibility.^28^, ^29^, ^30^ Our study demonstrated that TMT‐A performance is correlated with increased tau retention in the occipital lobe, whereas TMT‐B performance is correlated with tau retention in the parietal and occipital lobes in the AD continuum group. These correlations in the occipital lobe suggest an association with visual function, possibly indicative of the visual apraxia seen in AD. Although executive function deficits are often associated with frontal lobe dysfunction,^31^ a meta‐analysis showed that the Stroop test activates a broader network, including the middle frontal gyrus, anterior cingulate, parietal lobe regions, motor areas, and temporal lobe. This suggests that executive functions are not solely linked to frontal lobe activity.^32^ Furthermore, functional networks involved in executive function connect to the dorsolateral prefrontal cortex and inferior parietal lobe,^33^ indicating that tau protein deposition may disrupt neuronal stability and alter brain network connectivity. The JART, a Japanese version of the National Adult Reading Test (NART), estimates the premorbid intelligence quotients (IQs) of patients with dementia.^34^ The IQs derived using the NART are generally unaffected by cognitive impairment.^35^, ^36^, ^37^ However, some studies have shown significant impairment of NART performance in patients with AD.^38^, ^39^ Our results indicated that tau retention in the left temporal lobe was associated with the JART scores, suggesting that the premorbid IQs derived using the JART are affected by cognitive impairment.

In the non‐AD tauopathy group, the regional pattern of tau deposition and its correlation with cognitive function differed from those observed in the AD continuum group, despite both groups undergoing the same neuropsychological tests. For example, tau tracer retention in the left frontal lobe correlated with the MMSE scores in the non‐AD tauopathy group, which aligns with the characteristics of FTLD, as this group predominantly comprised patients with FTLD. FTLD primarily affects the frontal lobe, including Broca's area. In contrast, the AD continuum group showed fewer correlations in the frontal lobe, as most patients had Braak stages below IV to V. Braak stages correlate with clinical symptoms and disease severity, and patients in the most severe stages were under‐represented in this study.^7^, ^8^, ^40^ These differences suggest that parietal and occipital lobe function influences neuropsychological scores in AD, whereas frontal lobe function plays a more significant role in non‐AD tauopathies. Further research is necessary to clarify the relationship between tau deposition and neuropsychological functioning, particularly within each subtype of non‐AD tauopathies.

The item‐by‐item analysis of the ADAS‐Cog and MMSE revealed distinct correlation patterns. In the AD continuum group, compared to tau tracer retention in the right temporoparietal lobe, that in the left temporoparietal lobe showed a stronger correlation with immediate and delayed recall of the ADAS‐Cog, consistent with previous in vivo studies.^14^ Performance on the task of naming objects and fingers of the ADAS‐Cog correlated with tracer retention in the left frontal and bilateral temporal lobes only in the non‐AD tauopathy group. The Broca's area and temporal cortex are crucial for naming objects,^41^, ^42^, ^43^ and patients with FTLD exhibit more severe naming impairments, compared to those with AD.^21^ Tau retention in the frontal and temporal lobes may impair this function. ADAS‐Cog constructional praxis and MMSE copy design performances correlated with tau retention in the left occipital and posterior parietal lobes in the AD continuum group, though prior research indicated stronger correlations with the right hemisphere for visuospatial functions.^22^ In contrast, MMSE copy design performance of the non‐AD tauopathy group correlated with tracer retention in the right temporal lobe, suggesting that these neuropsychological tests may involve regions beyond those traditionally linked to visuospatial processing. Previous MRI and nuclear perfusion studies have shown that performance on many MMSE items rely on left hemispheric cognitive processing.^44^, ^45^, ^46^ Tau PET studies also revealed that the MMSE scores were negatively correlated with tau SUVR in the left anterior temporal cortex in patients with AD.^22^ Our findings also demonstrated a predominant left‐sided correlation between MMSE performance and tau tracer retention, consistent with previous studies. Performance on MMSE items such as calculation, command following, and sentence repetition exhibited stronger associations with tau tracer retention in the left posterior cortical areas within the AD continuum group. Functional MRI studies have shown a more prominent activation of the left hemisphere when solving arithmetic problems, compared to the right hemisphere.^47^ Regarding sentence repetition, the left hemisphere demonstrated a higher correlation than the right hemisphere did, in both groups. The left perisylvian core, including the arcuate fasciculus, has been implicated in repetition.^48^, ^49^ However, voxel‐based MRI analyses have identified the left posterior temporo‐parietal cortex, rather than the arcuate fasciculus, as critical for repetition.^50^ These findings suggest that a broader network of brain regions influences MMSE performance.

This study has some limitations. First, as Aβ and tau pathology was determined solely through 18F‐florbetaben and florzolotau PET imaging, without neuropathological confirmation, the possibility of false‐positive or false‐negative results cannot be excluded. However, previous neuropathological studies have shown that 18F‐florbetaben PET has high sensitivity (97.9%) and specificity (88.9%) for detecting histopathology‐confirmed neuritic Aβ plaques.^51^ Second, although florzolotau (18F) is considered a promising tracer for detecting tau fibrils, especially in four‐repeat tauopathies,^15^, ^17^, ^52^ few studies have used this tracer, and pathological proof remains limited. Consequently, the potential for off‐target binding is still uncertain and requires further investigation. Third, non‐AD tauopathies are highly heterogeneous. Although florzolotau (18F) cannot distinguish between three‐repeat/four‐repeat tau isoforms of primary tauopathy and mixed 3+4‐repeat NFTs in AD and PART, the latter, where tau pathology accumulates in the medial temporal lobe, was considered a non‐AD tauopathy in this study. Future research should compare PART to other FTLD‐tau subtypes. Fourth, as this study was conducted at a single center, with a racially and ethnically homogeneous sample, these institution‐specific factors may limit the generalizability of the findings.

The present study identified relationships between local tau pathology and neuropsychological test performance in patients with tauopathy. These findings enhance our understanding of tau protein deposition in tauopathies and highlight the significance of tau PET imaging in revealing the mechanisms of cognitive impairment.

## CONFLICT OF INTEREST STATEMENT

D.I. has received honoraria from Daiichi Sankyo, Nihon Medi‐Physics, Kowa, PDRadiopharma, Otsuka Pharmaceutical, Lilly, and Eisai and has a joint research agreement with Sysmex. The other authors declare that they have no conflicts of interest.

## CONSENT STATEMENT

The study protocol and design were approved by the Keio University School of Medicine Ethics Committee (#N20170237), with informed consent obtained from the participants or their caregivers. Approval for the florzolotau (18F) study was granted by the Radiation Drug Safety Committee and the National Institutes for Quantum Science and Technology Certified Review Board of Japan. This study adheres to the ethical standards of the Declaration of Helsinki.

## Supporting information



Supporting Information

Supporting Information

## References

[1] Wang Y , Mandelkow E . Tau in physiology and pathology. Nat Rev Neurosci. 2016;17:5‐21. 26631930 10.1038/nrn.2015.1

[2] Leuzy A , Chiotis K , Lemoine L , et al. Tau PET imaging in neurodegenerative tauopathies—still a challenge. Mol Psychiatry. 2019;24:1112‐1134. 30635637 10.1038/s41380-018-0342-8 PMC6756230

[3] Barker WW , Luis CA , Kashuba A , et al. Relative frequencies of Alzheimer disease, Lewy body, vascular and frontotemporal dementia, and hippocampal sclerosis in the State of Florida Brain Bank. Alzheimer Dis Assoc Disord. 2002;16:203‐212. 12468894 10.1097/00002093-200210000-00001

[4] Barnes DE , Yaffe K . The projected effect of risk factor reduction on Alzheimer's disease prevalence. Lancet Neurol. 2011;10:819‐828. 21775213 10.1016/S1474-4422(11)70072-2 PMC3647614

[5] Zhang H , Wei W , Zhao M , et al. Interaction between Aβ and tau in the pathogenesis of Alzheimer's disease. Int J Biol Sci. 2021;17:2181‐2192. 34239348 10.7150/ijbs.57078 PMC8241728

[6] Dickson DW , Kouri N , Murray ME , Josephs KA . Neuropathology of frontotemporal lobar degeneration‐tau (FTLD‐tau). J Mol Neurosci. 2011;45:384‐389. 21720721 10.1007/s12031-011-9589-0 PMC3208128

[7] Braak H , Braak E . Neuropathological stageing of Alzheimer‐related changes. Acta Neuropathol. 1991;82:239‐259. 1759558 10.1007/BF00308809

[8] Braak H , Braak E . Staging of Alzheimer's disease‐related neurofibrillary changes. Neurobiol Aging. 1995;16:271‐284. 7566337 10.1016/0197-4580(95)00021-6

[9] Brown CA , Das SR , Cousins KAQ , et al. Tau burden is best captured by magnitude and extent: tau‐MaX as a measure of global tau. Alzheimers Dement. 2025;21:e70346. 40613479 10.1002/alz.70346 PMC12231225

[10] La Joie R , Visani AV , Baker SL , et al. Prospective longitudinal atrophy in Alzheimer's disease correlates with the intensity and topography of baseline tau‐PET. Sci Transl Med. 2020;12:eaau5732. 31894103 10.1126/scitranslmed.aau5732 PMC7035952

[11] Malpas CB , Sharmin S , Kalincik T . The histopathological staging of tau, but not amyloid, corresponds to antemortem cognitive status, dementia stage, functional abilities and neuropsychiatric symptoms. Int J Neurosci. 2021;131:800‐809. 32303140 10.1080/00207454.2020.1758087

[12] Nelson PT , Alafuzoff I , Bigio EH , et al. Correlation of Alzheimer disease neuropathologic changes with cognitive status: a review of the literature. J Neuropathol Exp Neurol. 2012;71:362‐381. 22487856 10.1097/NEN.0b013e31825018f7 PMC3560290

[13] Guillozet AL , Weintraub S , Mash DC , Mesulam MM . Neurofibrillary tangles, amyloid, and memory in aging and mild cognitive impairment. Arch Neurol. 2003;60:729‐736. 12756137 10.1001/archneur.60.5.729

[14] Ossenkoppele R , Smith R , Ohlsson T , et al. Associations between tau, Aβ, and cortical thickness with cognition in Alzheimer disease. Neurology. 2019;92:e601‐e612. 30626656 10.1212/WNL.0000000000006875 PMC6382060

[15] Tagai K , Ono M , Kubota M , et al. High‐contrast in vivo imaging of tau pathologies in Alzheimer's and non‐Alzheimer's disease tauopathies. Neuron. 2021;109:42‐58.e8. 33125873 10.1016/j.neuron.2020.09.042

[16] Yagi T , Ito D , Sugiyama D , et al. Diagnostic accuracy of neuropsychological tests for classification of dementia. Neurol Asia. 2016;21:47‐54.

[17] Mashima K , Ito D , Kameyama M , et al. Extremely low prevalence of amyloid positron emission tomography positivity in Parkinson's disease without dementia. Eur Neurol. 2017;77:231‐237. 28285306 10.1159/000464322

[18] Seibyl J , Catafau AM , Barthel H , et al. Impact of training method on the robustness of the visual assessment of 18F‐Florbetaben PET scans: results from a phase‐3 study. J Nucl Med. 2016;57:900‐906. 26823561 10.2967/jnumed.115.161927

[19] Arriagada PV , Growdon JH , Hedley‐Whyte ET , Hyman BT . Neurofibrillary tangles but not senile plaques parallel duration and severiti of Alzheimer's disease. Neurology. 1992;42:631‐639. 1549228 10.1212/wnl.42.3.631

[20] Cho H , Choi JY , Hwang MS , et al. Tau PET in Alzheimer disease and mild cognitive impairment. Neurology. 2016;87:375‐383. 27358341 10.1212/WNL.0000000000002892

[21] Bejanin A , Schonhaut DR , La Joie R , et al. Tau pathology and neurodegeneration contribute to cognitive impairment in Alzheimer's disease. Brain. 2017;140:3286‐3300. 29053874 10.1093/brain/awx243 PMC5841139

[22] Ossenkoppele R , Schonhaut DR , Schöll M , et al. Tau PET patterns mirror clinical and neuroanatomical variability in Alzheimer's disease. Brain. 2016;139:1551‐1567. 26962052 10.1093/brain/aww027 PMC5006248

[23] Bullich S , Mueller A , De Santi S , et al. Evaluation of tau deposition using 18F‐PI‐2620 PET in MCI and early AD subjects—a MissionAD tau sub‐study. Alzheimers Res Ther. 2022;14:105. 35897078 10.1186/s13195-022-01048-x PMC9327167

[24] Johnson KA , Schultz A , Betensky RA , et al. Tau positron emission tomographic imaging in aging and early Alzheimer disease. Ann Neurol. 2016;79:110‐119. 26505746 10.1002/ana.24546 PMC4738026

[25] Rabin LA , Barr WB , Burton LA . Assessment practices of clinical neuropsychologists in the United States and Canada: a survey of INS, NAN, and APA Division 40 members. Arch Clin Neuropsychol. 2005;20:33‐65. 15620813 10.1016/j.acn.2004.02.005

[26] Clark RE . Current topics regarding the function of the medial temporal lobe memory system. Curr Top Behav Neurosci. 2018;37:13‐42. 29589322 10.1007/7854_2017_481

[27] MacPherson SE , Cox SR , Dickie DA , et al. Processing speed and the relationship between Trail Making Test‐B performance, cortical thinning and white matter microstructure in older adults. Cortex. 2017;95:92‐103. 28865241 10.1016/j.cortex.2017.07.021 PMC5637162

[28] Arbuthnott K , Frank J . Trail making test, part B as a measure of executive control: validation using a set‐switching paradigm. J Clin Exp Neuropsychol. 2000;22:518‐528. 10923061 10.1076/1380-3395(200008)22:4;1-0;FT518

[29] Kortte KB , Horner MD , Windham WK . The trail making test, part B: cognitive flexibility or ability to maintain set? Appl Neuropsychol. 2002;9:106‐109. 12214820 10.1207/S15324826AN0902_5

[30] Corrigan JD , Hinkeldey NS . Relationships between parts A and B of the trail making test. J Clin Psychol. 1987;43:402‐409. 3611374 10.1002/1097-4679(198707)43:4<402::aid-jclp2270430411>3.0.co;2-e

[31] Stuss DT , Levine B , Alexander MP , et al. Wisconsin card sorting test performance in patients with focal frontal and posterior brain damage: effects of lesion location and test structure on separable cognitive processes. Neuropsychologia. 2000;38:388‐402. 10683390 10.1016/s0028-3932(99)00093-7

[32] Alvarez JA , Emory E . Executive function and the frontal lobes: a meta‐analytic review. Neuropsychol Rev. 2006;16:17‐42. 16794878 10.1007/s11065-006-9002-x

[33] Yeager BE , Bruss J , Duffau H , et al. Central precuneus lesions are associated with impaired executive function. Brain Struct Funct. 2022;227:3099‐3108. 36087124 10.1007/s00429-022-02556-0 PMC9743014

[34] Matsuoka K , Uno M , Kasai K , Koyama K , Kim Y . Estimation of premorbid IQ in individuals with Alzheimer's disease using Japanese ideographic script (Kanji) compound words: Japanese version of National Adult Reading Test. Psychiatry Clin Neurosci. 2006;60:332‐339. 16732750 10.1111/j.1440-1819.2006.01510.x

[35] O'Carroll RE , Baikie EM , Whittick JE . Does the National Adult Reading Test hold in dementia? Br J Clin Psychol. 1987;26:315‐316. 3427255 10.1111/j.2044-8260.1987.tb01367.x

[36] Law R , O'Carroll RE . A comparison of three measures of estimating premorbid intellectual level in dementia of the Alzheimer type. Int J Geriatr Psychiatry. 1998;13:727‐730. 9818309 10.1002/(sici)1099-1166(1998100)13:10<727::aid-gps851>3.0.co;2-2

[37] Crawford JR , Besson JA , Parker DM , Sutherland KM , Keen PL . Estimation of premorbid intellectual status in depression. Br J Clin Psychol. 1987;26:313‐314. 3427254 10.1111/j.2044-8260.1987.tb01366.x

[38] Stebbins GT , Wilson RS , Gilley DW , Bernard BA , Fox JH . Use of the National Adult Reading Test to estimate premorbid IQ in dementia. Clin Neuropsychol. 1990;4:18‐24. 29022436 10.1080/13854049008401493

[39] Frick A , Wahlin T‐BR , Pachana NA , Byrne GJ . Relationships between the National Adult Reading Test and memory. Neuropsychology. 2011;25:397‐403. 21401261 10.1037/a0021988

[40] Braak H , Thal DR , Ghebremedhin E , Del Tredici K . Stages of the pathologic process in Alzheimer disease: age categories from 1 to 100 years. J Neuropathol Exp Neurol. 2011;70:960‐969. 22002422 10.1097/NEN.0b013e318232a379

[41] Cappa SF , Binetti G , Pezzini A , Padovani A , Rozzini L , Trabucchi M . Object and action naming in Alzheimer's disease and frontotemporal dementia. Neurology. 1998;50:351‐355. 9484352 10.1212/wnl.50.2.351

[42] Xu Z , Shen B , Taji W , Sun P , Naya Y . Convergence of distinct functional networks supporting naming and semantic recognition in the left inferior frontal gyrus. Hum Brain Mapp. 2020;41:2389‐2405. 32065445 10.1002/hbm.24953 PMC7268040

[43] Lubrano V , Filleron T , Démonet J‐F , Roux F‐E . Anatomical correlates for category‐specific naming of objects and actions: a brain stimulation mapping study. Hum Brain Mapp. 2014;35:429‐443. 23015527 10.1002/hbm.22189 PMC6869226

[44] Iwabuchi Y , Shiga T , Kameyama M , et al. Striatal dopaminergic depletion pattern reflects pathological brain perfusion changes in Lewy body diseases. Mol Imaging Biol. 2022;24:950‐958. 35701723 10.1007/s11307-022-01745-x PMC9681681

[45] Apostolova LG , Lu PH , Rogers S , et al. 3D mapping of mini‐mental state examination performance in clinical and preclinical Alzheimer disease. Alzheimer Dis Assoc Disord. 2006;20:224‐231. 17132966 10.1097/01.wad.0000213857.89613.10

[46] Lampl Y , Sadeh M , Laker O , Lorberboym M . Correlation of neuropsychological evaluation and SPECT imaging in patients with Alzheimer's disease. Int J Geriatr Psychiatry. 2003;18:288‐291. 12673603 10.1002/gps.827

[47] Abd Hamid AI , Yusoff AN , Mukari SZ‐MS , Mohamad M . Brain activation during addition and subtraction tasks in‐noise and in‐quiet. Malays J Med Sci. 2011;18:3‐15. 22135581 PMC3216211

[48] Wang H , Li S , Dai Y , Yu Q . Correlation between speech repetition function and the Arcuate Fasciculus in the dominant hemisphere detected by Diffusion Tensor Imaging tractography in stroke patients with aphasia. Med Sci Monit. 2020;26:e928702. 33277460 10.12659/MSM.928702 PMC7724775

[49] Berthier ML , Starkstein SE , Leiguarda R , et al. Transcortical aphasia. Importance of the nonspeech dominant hemisphere in language repetition. Brain. 1991;114 (Pt 3):1409‐1427. 2065258 10.1093/brain/114.3.1409

[50] Baldo JV , Katseff S , Dronkers NF . Brain regions underlying repetition and auditory‐verbal short‐term memory deficits in aphasia: evidence from voxel‐based lesion symptom mapping. Aphasiology. 2012;26:338‐354. 24976669 10.1080/02687038.2011.602391 PMC4070523

[51] Sabri O , Sabbagh MN , Seibyl J , et al. Florbetaben PET imaging to detect amyloid beta plaques in Alzheimer's disease: phase 3 study. Alzheimers Dement. 2015;11:964‐974. 25824567 10.1016/j.jalz.2015.02.004

[52] Shimohama S , Tezuka T , Takahata K , et al. Impact of amyloid and tau PET on changes in diagnosis and patient management. Neurology. 2023;100:E264‐E274. 36175151 10.1212/WNL.0000000000201389

