# Supplementary material for: Florzolotau (18F) retention is linked to neuropsychological performance in tauopathy
Source: Alzheimers Dement. 2026 Apr 1;22(4):e71319. doi: 10.1002/alz.71319 (PMC13045494; doi:10.1002/alz.71319)
Supplement: Supplementary file 1 — Supporting Information [file ALZ-22-e71319-s002.pdf]

Supplementary Table 1. Inclusion criteria

	HC	MCI	AD	Non-AD dementia
Age (y)	40–85			
Number of years of education (y)	>12			
MMSE score	24–30		<23	-
CDR score	0	0.5	0.5–1	-
ADL	Normal	Normal to almost normal	-	-
GDS score	<6	-		
CES-D score	<16	-		

Abbreviations: AD, Alzheimer's disease; ADL, activities of daily living; CDR, Clinical Dementia Rating; CES-D, The Center for Epidemiologic Studies Depression Scale; GDS, Geriatric Depression Scale; HC, healthy control; MCI, mild cognitive impairment; MMSE, Mini-Mental State Examination

Supplementary Table 2. Exclusion and discontinuance criteria

Criteria	Details
Exclusion criteria	History of schizophrenia, major depression, or bipolar disorder, as defined by the DSM-5, in the previous year
	Brain tumors, normal pressure hydrocephalus, multiple cerebral infarctions, seizure disorder, epilepsy, subdural hematoma, Huntington disease, or multiple sclerosis
	Alcoholic or drug addiction, as defined by the DSM-5, in the previous 2 years
	Receipt of 18F-PI-2620 tau PET
	Focal brain lesions caused by infectious diseases or cerebral infarction affecting cognitive function identified on the screening MRI
	Not having undergone structural MRI and tau PET within the same 6-month period
	Severe systemic disease or unstable condition
	Syphilis or thyroid disorders affecting cognitive function
	MRI contraindications (metals in the body or claustrophobia)
	Symptoms that make it difficult to adhere to protocols
	Participation in a drug clinical trial
Discontinuance criteria	Difficulty in continuing the trial because of worsening complications or adverse events.
	The requirements not being met following registration.
	Withdrawal of consent
	Termination of the entire study

Abbreviations: DSM-5, Diagnostic and Statistical Manual of Mental Disorders, 5th Edition; PET, positron emission tomography; MRI, magnetic resonance imaging

Supplementary Fig.1 Correlation between tau tracer retention and ADAS-cog items

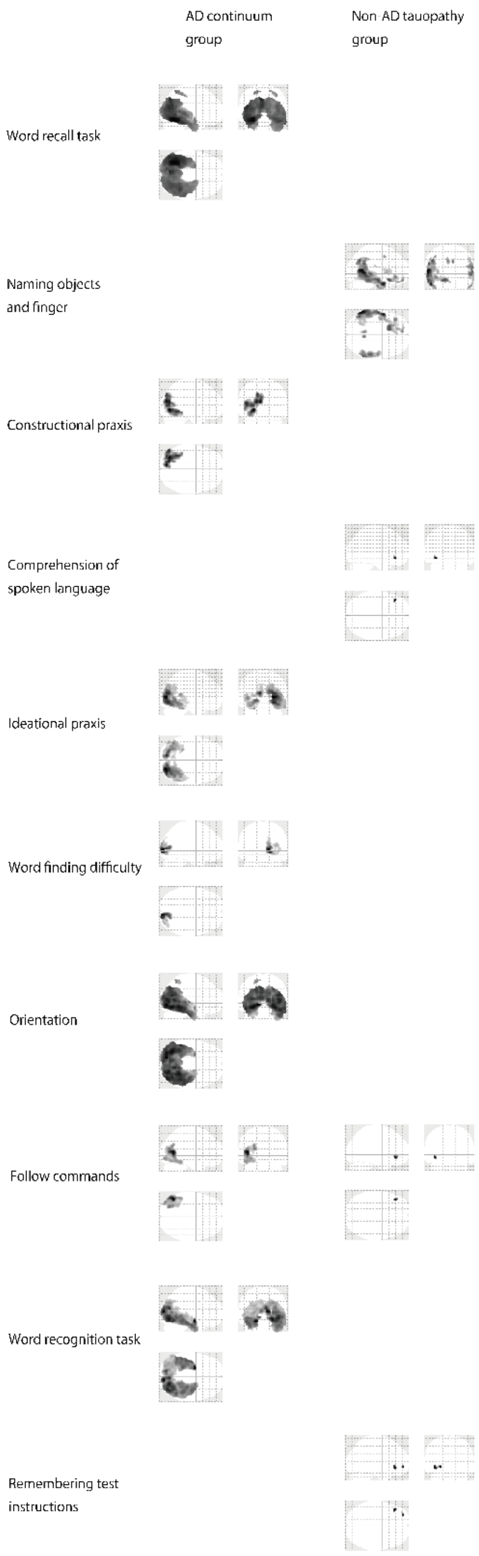

Note. Several ADAS-cog items showed a correlation with tau tracer retention in both the AD continuum and non-AD tauopathy groups. The empty area in the figure indicates that there is no significant difference.

Abbreviations: AD, Alzheimer's disease; ADAS-cog, AD Assessment Scale-Cognitive Subscale

Supplementary Fig.2 Correlation between tau tracer retention and MMSE items

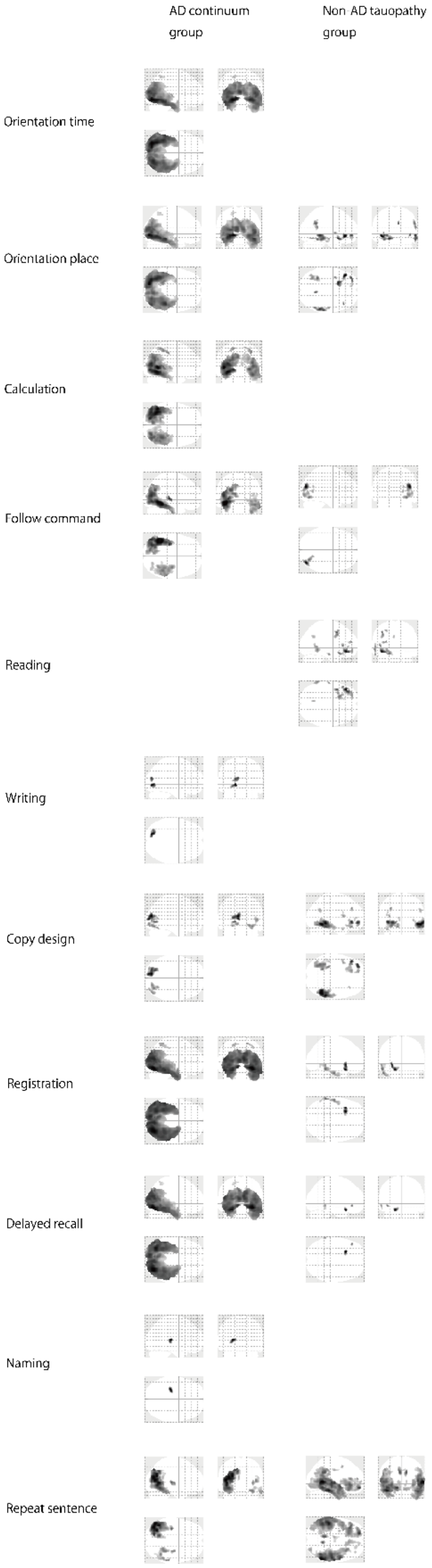

Note. Several MMSE items showed a correlation with tau tracer retention in both the AD continuum and non-AD tauopathy groups. The empty area in the figure indicates that there is no significant difference.

Abbreviations: AD, Alzheimer’s disease; MMSE, Mini-Mental State Examination
